# Correction: Efficacy and Safety of Patritumab Deruxtecan (HER3-DXd) in EGFR Inhibitor–Resistant, *EGFR*-Mutated Non–Small Cell Lung Cancer

**DOI:** 10.1158/2159-8290.CD-22-0365

**Published:** 2022-06-02

In Table 1 in the original version of this article (1), the value in row “Ex19ins” and column “Pooled RDE” should be “1 (2).” Table 1 has been corrected in the latest online HTML and PDF versions of the article. The authors regret the error.
